# Evaluating Carbonate System Algorithms in a Nearshore System: Does Total Alkalinity Matter?

**DOI:** 10.1371/journal.pone.0165191

**Published:** 2016-11-28

**Authors:** Jonathan M. Jones, Julia Sweet, Mark A. Brzezinski, Heather M. McNair, Uta Passow

**Affiliations:** 1 Marine Science Institute, University of California Santa Barbara, Santa Barbara, California, United States of America; 2 Cabrillo National Monument, San Diego, California, United States of America; 3 Department of Ecology, Evolution and Marine Biology, University of California Santa Barbara, Santa Barbara, California, United States of America; Auckland University of Technology, NEW ZEALAND

## Abstract

Ocean acidification is a threat to many marine organisms, especially those that use calcium carbonate to form their shells and skeletons. The ability to accurately measure the carbonate system is the first step in characterizing the drivers behind this threat. Due to logistical realities, regular carbonate system sampling is not possible in many nearshore ocean habitats, particularly in remote, difficult-to-access locations. The ability to autonomously measure the carbonate system *in situ* relieves many of the logistical challenges; however, it is not always possible to measure the two required carbonate parameters autonomously. Observed relationships between sea surface salinity and total alkalinity can frequently provide a second carbonate parameter thus allowing for the calculation of the entire carbonate system. Here, we assessed the rigor of estimating total alkalinity from salinity at a depth <15 m by routinely sampling water from a pier in southern California for several carbonate system parameters. Carbonate system parameters based on measured values were compared with those based on estimated TA values. Total alkalinity was not predictable from salinity or from a combination of salinity and temperature at this site. However, dissolved inorganic carbon and the calcium carbonate saturation state of these nearshore surface waters could both be estimated within on average 5% of measured values using measured pH and salinity-derived or regionally averaged total alkalinity. Thus we find that the autonomous measurement of pH and salinity can be used to monitor trends in coastal changes in DIC and saturation state and be a useful method for high-frequency, long-term monitoring of ocean acidification.

## Introduction

Carbon dioxide (CO_2_) levels are rising globally in the atmosphere as a result of anthropogenic influences such as the combustion of fossil fuels and manufacturing of cement [1]. As CO_2_ increases in the atmosphere, it equilibrates with the surface ocean causing an increase in the concentration of H^+^ ions and a reciprocal decrease in ocean pH. Termed ocean acidification (OA), this decrease in ocean pH and associated changes in carbonate speciation can be detrimental to the survival and development of many marine invertebrates both in pelagic [2–4] and benthic environments [5–7].

In order to adequately monitor ocean acidification in the California Current, the California Current Ocean Acidification Network recommends a combination of high frequency temporal monitoring and spatial mapping, especially in nearshore areas [8]. Collecting high frequency temporal data at fixed locations is often logistically difficult due to coastal access, instrumentation costs, and sample processing costs and spatial mapping of pH across ocean biomes [9, 10] is challenging due to high variability [11]. This variability, especially at biologically active depths 0–15 m [12], makes the carbonate system difficult to predict, a considerable challenge for established intertidal, estuarine, and some nearshore monitoring programs.

A set of four equations constrains the marine carbonate system: two equilibrium equations, a mass balance equation for total dissolved inorganic carbon (DIC) and a charge balance equation for total alkalinity (TA). The marine carbonate system can be quantitatively characterized by determining at least two of several measurable carbonate parameters, which include DIC, TA, partial pressure of CO_2_ (*p*CO_2_), and pH. Each of these parameters is measured separately but with any two of the four, the others may be calculated [13]. A series of autonomous underwater instruments have been developed to enhance the collection frequency and spatial resolution of pH measurements across a wide variety of marine biomes [9, 14–16]. These instruments permit continuous *in-situ* pH measurements, one of the four measureable carbonate system parameters. *p*CO_2_ can also be accurately measured *in situ* using autonomous instrumentation [17, 18]. While the remaining carbonate parameters can be calculated from measurements of pH and *p*CO_2_, this particular combination is susceptible to large calculation error derived from the measurement error for each respective parameter [19]. Thus the carbonate system can more accurately be constrained by including TA or DIC in the calculation.

TA is a quasi-conservative parameter of the carbonate system that can be estimated in some instances with site-specific algorithms from salinity alone or from a combination of salinity and temperature [12, 18, 20–22]. Including temperature in the prediction of TA can provide a proxy for upwelling-induced changes and increase TA predictability regionally. In oceanic regions where the contribution of biology to the carbonate system is relatively minor, salinity is the largest driver of TA [20, 23]. Coastal zones experience many factors in addition to changes in salinity from freshwater inputs that can drive TA variability thus making it more difficult to estimate TA. The production and export or dissolution of CaCO_3_, primary production and respiration [24, 25], nutrient cycling, and upwelling can all additively or synergistically alter TA in these systems. Organic alkalinity from biological productivity may also contribute significantly to TA in coastal zones [26].

Despite the complex nature of coastal environments, Cullison Gray and co-authors [18] were able to constrain autonomous *in situ* measurements of *p*CO_2_ in Monterey Bay on a buoy located ~8 km offshore on the 70 m isobath (36.83° N, 121.90° W) by estimating TA from salinity. In that study, measured pH and estimated TA from salinity were used to calculate *p*CO_2_ using the computer program CO2SYS. No bottle samples were taken to validate estimated TA, but measured *p*CO_2_ and *p*CO_2_ calculated from measured pH and estimated TA agreed well throughout. Cullison Gray et al. [18] concluded that the magnitude and variability in calculated *p*CO_2_ is driven principally by measured pH and not TA as calculated *p*CO_2_ was relatively insensitive to a fixed value for TA. There are instances, however, when calculated *p*CO_2_ significantly over-estimates measured *p*CO_2_ [27].

Here, we present a dataset of three carbonate system parameters measured weekly over a two-year period to detect whether TA estimated from salinity or salinity and temperature is (1) a reliable predictor of measured TA and (2) a useful parameter for calculating the carbonate system at depths <15 m in Southern California. Discrete water samples were collected between August 2012 and March 2014 at Stearns Wharf, a long-term monitoring station of the Santa Barbara Coastal LTER in Santa Barbara, CA and analyzed for DIC, pH, TA, and salinity. We then compared TA estimated (TA_est_) from sea surface salinity (SSS) and sea surface temperature (SST) or SSS using several published algorithms [12, 18, 20–22] to TA measured or calculated from the collected bottle samples (TA_lab_). The nearest model region for each algorithm was used for the calculations and measured TA and salinity from our site were also compared. The effect of TA_est_ as a contributing parameter to carbonate system calculations was then examined to determine if having one measured and one estimated parameter would introduce significant error in the calculated values of DIC or calcium carbonate saturation state.

## Methods

### Sample collection

Carbonate system parameters were sampled weekly from 1 August, 2012 through 14 March, 2014 through a hatch in the pier at Stearns Wharf, Santa Barbara, (34.4107°N 119.6874°W). No permissions were required to collect water samples from a public pier and no endangered or protected species were involved in the field sampling procedures. Water samples were collected using a 627 mm long, 1.7 L Go-Flo bottle. Exact sampling depth was initially determined via SCUBA to align the sampling bottle directly beside a moored CTD. Samples were collected, prepared, and processed following “Guide to Best Practices for Ocean CO_2_ Measurements” [28]. Specifically, samples were transferred directly from the Go-Flo bottle to acid-washed 380 mL brown glass beer bottles or 250 mL Pyrex borosilicate bottles with care to minimize bubbling. Brown glass bottles were used for pH and DIC samples [29] and Pyrex bottles were used for all TA analysis [28]. All samples were overfilled by a minimum of 50% volume leaving ~1% headspace and fixed with 60–110 μL of saturated mercuric chloride solution [28] depending on bottle volume. Samples were sealed and stored at 2°C until analysis. A subset of unfixed samples was collected and pH_T_ measured within two hours of collection.

### Quality Control

Quality assurance was achieved at multiple levels. Replicate bottle samples were collected from the same Go-Flo bottle, treated with the same HgCl_2,_ and analyzed both at Scripps Institute of Oceanography (Dickson lab) and at the University of California Santa Barbara (UCSB, Passow lab). After the initial year of sampling (n = 40 samples), samples were analyzed at UCSB only. In addition to direct comparison between laboratories, performance for the DIC instrument at UCSB was tested during an intercalibration exercise in December 2013 [30]. DIC measured at UCSB was on average ± 0.02% within target concentrations.

### Discrete Measurements

DIC and pH were measured from all 70 samples. Direct measurements of TA were only conducted from samples during the first year. pH was measured spectrophotometrically at UCSB using unpurified m-cresol purple dye at standard temperature (25°C) and reported on the total hydrogen ion concentration scale (pH_T_). Scripps measured pH_T_ spectrophotometrically at 25°C on the total hydrogen ion concentration scale using purified m-cresol dye. Although measurement methodology between laboratories was consistent, the use of unpurified dye can introduce measurement errors as large as 0.02 pH units [31]. DIC was measured at UCSB by acidification and subsequent quantification of released CO_2_ using a LI-COR NDIR *p*CO_2_ analyzer. Certified reference materials (batches 108, 144; A. Dickson) were used as a control to validate instrument accuracy. Scripps determined DIC by way of vacuum extraction/manometric procedure. DIC samples (n = 23) were measured at both laboratories for samples collected between August 1, 2012 and June 3, 2013. All TA measurements (n = 37) were performed at Scripps using a two-stage, potentiometric, open-cell titration [32]. Following the first year, when TA was not directly measured, TA was calculated for each sample using paired DIC and pH_T_ measurements and the computer program CO2SYS. Program preferences were set to use carbonate system solubility products from Mehrbach et al., [33] refit by Dickson and Millero [34], K_HSO4-_ dissociation constants from Dickson et al., [35], and total boron from Uppstrom [36]. TA values used for the comparative analysis, designated (TA_lab_), reflect directly measured values where available and TA values calculated from measured DIC and measured pH_T_ when TA was not directly measured.

### Autonomous Measurements

SSS and SST were measured using a moored conductivity, temperature, and depth (CTD) instrument (SeaBird SBE16 plus). The CTD is located at an average water depth 4 m off the bottom and approximately 4 m from the surface depending on tidal height. (L. Washburn; Sea-surface water temperature, Santa Barbara Harbor, Santa Barbara, CA, USA, 1955 to present, ongoing DOI: 10.6073/pasta/ba67439ac372ec 12610488bc115a4b4e). Salinity was referenced against discrete water samples using a benchtop salinometer (YSI 3100) calibrated against a reference conductivity solution (YSI 3161).

### Auxiliary data

Upwelling and rainfall data were used to identify possible drivers of carbonate system variability at Stearns Wharf. Chlorophyll *a* and nutrient concentration data were collected weekly at the same station. Chlorophyll *a* was determined according to Parsons et al. [37] and nutrients were frozen until analysis on a Zellweger Analytics, Inc QuickChem 8000. Rainfall data were obtained from the County of Santa Barbara Public Works Historical Rainfall Information (Santa Barbara City College Station, 34.4059° N, 119.6973° W). Upwelling indices were provided by the NOAA Southwest Fisheries Science Center (Bakun Index Values from NOAA/NMFS/PFEG for: 33N 119W).

## Results and Discussion

Replicate samples measured within each laboratory agreed well for measurements of pH_T_ (average SD = 0.015), DIC (average SD = 4.0 μmol kg^-1^), and TA (average SD = 1.5 μmol kg^-1^). Replicate pH_T_ measurements between laboratories correlated highly significantly (r^2^ = 0.87, n = 31, p < 0.00001) with an average difference of 0.036. Replicate DIC samples between laboratories were also highly significantly correlated (r^2^ = 0.81, n = 23, p < 0.00001), as were measured and calculated TA (r^2^ = 0.89, n = 37, p < 0.00001) with average differences of 29.2 μmol kg^-1^ SW and 10.0 μmol kg^-1^ SW respectively. In the following it is assumed that all TA_lab_ values reflect the correct TA for that point in time and station.

**Fig 1:**
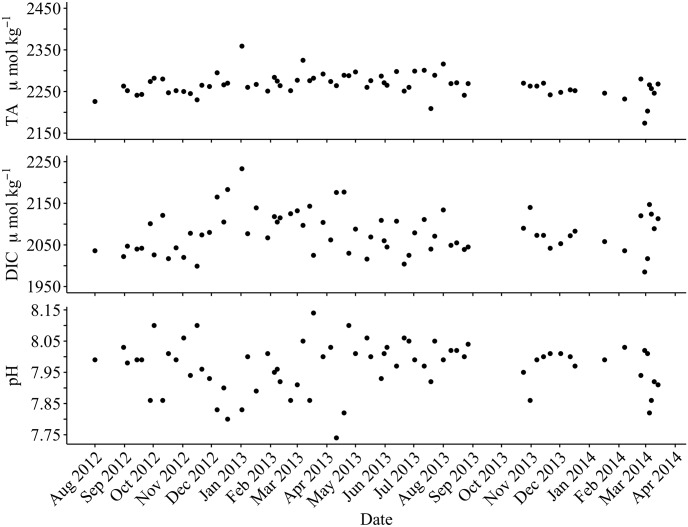
Carbonate system variability at Stearns Wharf. Variability in carbonate system parameters (TA, DIC, in situ pH) collected at Stearns Wharf between August 2012 and April 2014.

The entire carbonate system was markedly variable throughout the study period: pH_T_ ranged from 7.74 to 8.14, DIC from 1985 to 2223 μmol kg^-1^, and TA from 2174 to 2359 μmol kg^-1^ (Fig 1). The mean pH_T_ (mean = 7.96, SD = 0.08) at this site was lower than the global mean (8.05, [12, 18, 20–22]). DIC and pH are often variable in coastal regions due to the effects of upwelling, seasonal warming, and photosynthesis and respiration effects [11, 16]. TA, however, a quasi-conservative parameter, also varied by as much as 100 μmol kg^-1^ over a 7-day time frame in the absence of large freshwater inputs (Fig 1). Common sources of non-conservative TA variability that may account for the observed TA fluctuations include the accumulation of dissolved organic carbon, denitrification, and carbonate precipitation or dissolution [26]. Additional contributing influences specific to Stearns Wharf may include wave-driven resuspension of mineral-rich sediments and the addition of organics from activity on the pier, a common center for sport fishing and tourism. Such variable TA at Stearns Wharf without the accompanying fluctuation in salinity suggest that non-conservative influences at this site outweigh the conservative drivers of TA variability more commonly observed in open ocean and high rainfall regions.

**Fig 2:**
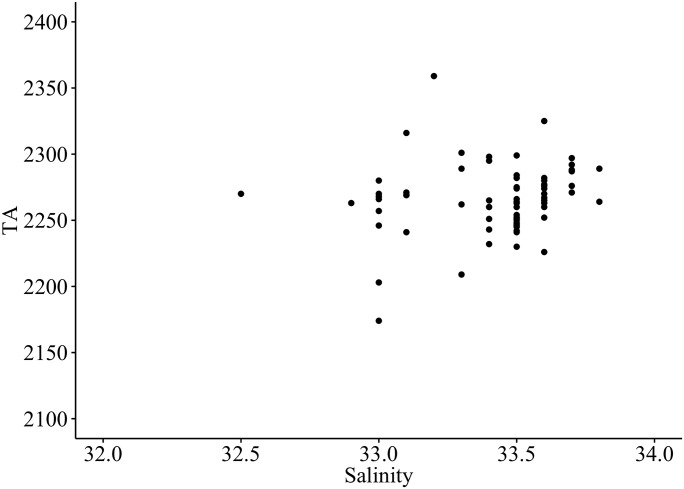
Relationship between salinity and total alkalinity (r^2^ = 0.014, n = 70).

There was no detectable relationship between TA_lab_ and SSS, with TA_lab_ fluctuating by as much as 150 μmol kg^-1^, and SSS by as little as 1 psu during the first year (Fig 2). While this type of variability may be expected for pH_T_ and DIC, it is not expected *a priori* for TA, which is often considered conservative with respect to temperature and pressure [38]. The greater Santa Barbara region experiences highly seasonal precipitation with pulses of rainfall during the late winter and early spring. A severe drought from 2012–2014 limited total rainfall to just 43.18 cm over the two-year study period. The greatest rainfall for any given day did not exceed five centimeters and the average rainfall per day was 0.08 cm. Precipitation and pulsed riverine influences did not likely contribute greatly to carbonate system variability at Stearns Wharf.

Biological production and upwelling or some combination of both, may potentially be responsible for the TA variability in our precipitation-limited coastal environment. Upwelling in the California Current brings cold, nutrient replete water to the surface fostering high primary productivity [39]. This nutrient-rich water has a relatively low pH and high DIC signature [40], but may also be indirectly responsible for a temporary increase in seawater TA due to negatively charged surface groups which can act as proton acceptors on phytoplankton and bacterial cells [41][20]. Cross and coauthors [26] reported TA variation due to combined organic production and denitrification on the order of ~15 μmol kg^-1^ SW, a potentially minor (<1%) contribution to the observed degree of variability at Stearns Wharf. Yang and coauthors found that organic-derived alkalinity could account for as much as a ~40 μmol kg^-1^ SW difference between measured and calculated TA in coastal waters for the Gulf of Mexico [42]. While there is undoubtedly some contribution of organic matter to TA variability at Stearns Wharf, the high correlation and relatively small average difference between measured and calculated TA values suggests that TA variability is not primarily driven by organic alkalinity at this site.

**Fig 3:**
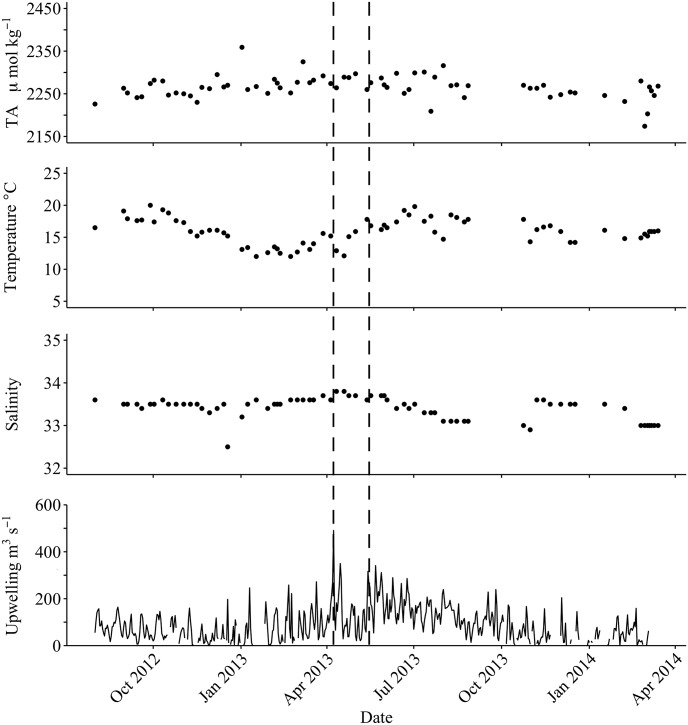
Upwelling as a driver of total alkalinity at Stearns Wharf. Total alkalinity (top) shows little connection to upwelling events, though there are occasional spikes in the upwelling index (dotted lines) that co-occur with changes in temperature and salinity.

Seasonal upwelling events visually appear to be correlated with TA variability at times, but upwelling did not exclusively drive carbonate chemistry dynamics during instances of TA fluctuation (Fig 3). Strong seasonal upwelling (~500 m^3^ s^-1^), observed in spring of 2013, was accompanied by a characteristic drop in water temperature (3°C), an increase in silicic acid (~6–12 μmol L^-1^), and an increase in chlorophyll (Chl a ~2–6 mg m^-3^). The carbonate system concurrently showed a marked response during this time: DIC increased by 100 μmol kg^-1^ and pH_T_ decreased by 0.20 units. TA, however, did not fluctuate during this period with a decrease of only 10 μmol kg^-1^. In addition to upwelling events that lacked a corresponding response in TA, there were also times when the carbonate system fluctuated substantially outside of upwelling periods (Fig 3). SSS and SST are often good indicators of upwelling, but in this case a multifactorial regression analysis with SST and SSS as the predictors showed no significant overall relationship with TA_lab_ (statistical package JMP 12, n = 70).

Estimates of TA rely on the existence of a relationship between SSS and TA. As expected from the absence of a relationship between TA_lab_ and SSS (r^2^ = 0.02 in this study), none of the known predictive equations for TA_est_ [12, 18, 20–22] demonstrated a statistically significant ability to predict TA (r^2^ = 0.02–0.03, slope = -0.03–0.12) at this site. Although these relationships are predominately used for open ocean environments and are less accurate at the shallow end of the depth range [12], Cullison Gray and coauthors [18] and Wootton and Pfister [21] successfully predicted carbonate system parameters using salinity-based relationships for coastal California (*p*CO_2_) and intertidal Washington (TA) respectively. Our findings show that in coastal systems TA cannot be predicted accurately from SSS or SSS + SST. Our study further reinforces previous findings [12] demonstrating that TA_est_ consistently underestimates true alkalinity (TA_lab_); at our station by on average 30 μmol kg^-1^, with a maximum of 66 μmol kg^-1^ and a minimum of 14 μmol kg^-1^.

Although estimates of TA from SSS or SSS combined with SST are not predictive of directly measured TA at this site, TA_est_ may be useful for estimating or constraining other parameters of the carbonate system. In one such study, *p*CO_2_ readings taken by an autonomous instrument were verified against calculated *p*CO_2_ using TA_est_ and directly observed pH [18]. A reasonable agreement between estimated and measured *p*CO_2_ suggests that any deviation due to errant TA_est_ did not significantly influence overall trends in calculated *p*CO_2_. This observation supports carbonate system insensitivity to changes in TA when directly measured pH is used as the second carbonate system parameter. While broad trends are visible, smaller scale fluctuations in TA are lost in such estimates. For example, an error of 100 μmol kg^-1^ in the estimate of TA leads to an error in DIC of ~ 90–95 μmol kg^-1^ or in pH_T_ of 0.14 to 0.17 units (depending on temperature), an uncertainty well beyond the acceptable error for measuring the carbonate system in the California Current [8].

**Fig 4:**
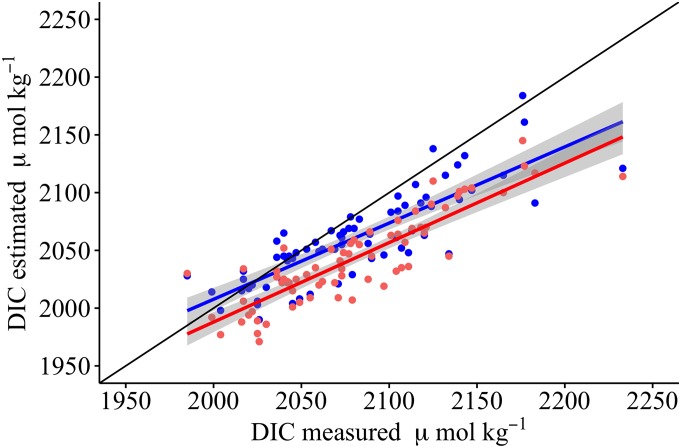
Relationship between measured and estimated DIC. DIC is estimated either from pH_T_ and TA_est_ (blue points) or pH_T_ and regional average TA (red points). The black line denotes the theoretical 1:1 relationship, the blue and red lines the respective correlations with the grey shading marking the 95% intervals. See text for details.

**Fig 5:**
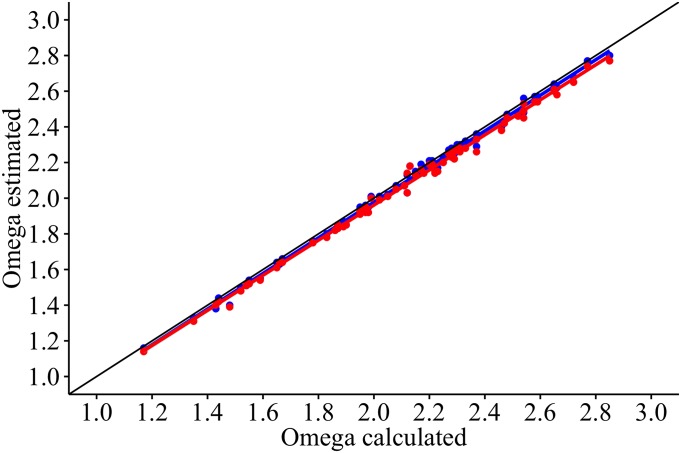
Relationship between calculated and estimated Ω_Arag_. The latter was approximated from measured pH_T_ and either TA_est_ (blue points and line, r^2^ = 0.996, y = 0.998x + 0.0271) or a regional average TA (red points and line, r^2^ = 0.996, y = 0.983x-0.0049). See text for details.

The Stearns Wharf data set was used to test for any significant difference between DIC measured from bottle samples and DIC estimated using TA_est_ with measured pH. Measured and estimated DIC values correlated well (r^2^ = 0.695, n = 70, p < 0.00001) demonstrating that overall trends in DIC can be estimated using TA_est_ (Fig 4), though the estimated values underestimate measured ones at higher DIC (>2025 μmol kg^-1^). We also tested how well DIC estimated from measured pH_T_ and a regional average TA (2222 μmol kg^-1^) could predict DIC. When combined with measured pH, calculated DIC using an average TA was more highly correlated to measured DIC (r^2^ = 0.760, n = 70, p < 0.00001) than when the SSS based estimated TA was used. Similarly, the saturation state for calcium carbonate in the form of aragonite, Ω_Arag_, could be equally well predicted (r^2^ = 0.995, n = 70, p < 0.00001) using either TA_est_ or an average TA (Fig 5) indicating the relative insensitivity of Ω_Arag_ to TA.

The data indicate that in some coastal environments, estimates of TA based on SSS or SST + SSS do not represent the true TA well and details of the carbonate system fluctuations are lost if such estimates are used. Moreover, TA may just as well be estimated from average values than from salinity. Regional averages of TA or salinity based estimates of TA may be effectively used to constrain other carbonate parameters and assess trends if paired with direct measurements of *p*CO_2_ or pH. Details of the fluctuations within the carbonate system will, however, be unresolved when TA is estimated. For long-term coastal monitoring programs where logistical restraints and frequent sampling are unachievable, estimating TA from SSS or SSS + SST may be a good option for obtaining a second carbonate system parameter. Our data indicate that although TA cannot be predicted well at a nearshore site in Southern California, DIC and Ω_Arag_ can be predicted to a reasonable level of accuracy. Furthermore, in areas that experience little precipitation, a constant regional average TA may work as well or better than that calculated from *in situ* salinity. Given the spatial and temporal heterogeneity of the coastal carbonate system, however, every site must be evaluated independently before a regional estimate or algorithm is applied.
